# Bold Suggestion by Smith

**DOI:** 10.1371/journal.pmed.0020226

**Published:** 2005-07-26

**Authors:** David Cohen

**Affiliations:** **1**Florida International UniversityMiami, FloridaUnited States of America

Richard Smith's key suggestion [1] is that medical journals “should stop publishing trials” and concentrate on “critically evaluating them.” This bold and radical suggestion deserves wide debate. It's obvious that many medical journals are losing relevance as vehicles for scientific information, but it's unclear what will save them. Even as journals strive to better enforce their conflicts-of-interest disclosure rules, drug companies will strive to find or create other publication outlets that can communicate to physicians precisely what advertisers wish to communicate. In sum, an unanticipated effect of purging clinical trial reports from medical journals might be an even larger proliferation of frank advertising outlets and messages that might more effectively catch doctors' attentions.
